# Investigation of Parallel and Orthogonal MIMO Antennas with Two-Notched Structures for Ultra-Wideband Application

**DOI:** 10.3390/mi14071406

**Published:** 2023-07-11

**Authors:** Liang Wang, Ziwei Li, Hongxing Zheng

**Affiliations:** School of Electronics and Information Engineering, Hebei University of Technology, Tianjin 300401, China; 202131903076@stu.hebut.edu.cn (L.W.); 201911901001@stu.hebut.edu.cn (Z.L.)

**Keywords:** coplanar waveguide feed, double-band notched, MIMO antenna, UWB antenna, isolation, orthogonal elements

## Abstract

Ultra-wideband (UWB) technology is widely used in many communication scenarios. However, narrowband systems can easily interfere with the UWB system, which generates multipath fading. In order to solve these interferences and meet the design requirements of high isolation of multiple-input multiple-output (MIMO) antennas, two MIMO antennas with double-notch structures are designed. Firstly, two U-shaped slots are etched on the radiating patch and feeder to achieve notch characteristics in WiMAX and ITU bands. Using this antenna element, a two-element antenna is put symmetrically in parallel, and two rectangular branches are loaded to improve the isolation. The size is 0.57*λ* × 0.32*λ* × 0.013*λ* (at 2.5 GHz). Then, a four-element antenna is designed to meet the requirements for another application; here, each element is placed orthogonally to each other, and the isolation is improved through loading a cross-shaped branch in the middle of these elements. The size is 0.57*λ* × 0.57*λ* × 0.013*λ*. Both antenna samples are tested to verify the design. Measurement results show that the working bandwidth is 2.45–14.88 GHz and 2.14–14.95 GHz, the isolation is greater than 17 and 20 dB, and the peak gain is 5.7 and 5.9 dBi for the two- and four-element MIMO antenna, respectively. Compared to the references, the designed antennas have a wider bandwidth and a higher gain and radiation efficiency. They are well-suited for diverse wireless applications.

## 1. Introduction

Since the application of ultra-wideband (UWB) frequency was proposed, it has become the focus of many engineers of wireless communication technology. Due to the high transmission rate and low power consumption, UWB technology has been widely used in the fields of ground-penetrating radar [1,2], wireless sensors [3], precise positioning [4], biomedical engineering [5,6], etc. However, the presence of many narrowband communication systems creates some interferences with the UWB system because those frequency bands are also included in the operating band of the UWB system. Examples are the Worldwide Interoperability for Microwave Access (WiMAX, 3.3–3.7 GHz), the International Telecommunication Union band (ITU, 8.01–8.5 GHz), etc. At present, the simplest method to filter the interference of the narrowband signals is designing an antenna with a band-notched property. To achieve the notch property, the antenna is equipped with etched slots [7,8], defected ground structures [9], and attached parasitic elements [10,11]. The advantages of those methods are a simple structure and easy design, and that the size of the antenna is not increased, all of which are conducive to the miniaturization and large-scale integration of the antenna. 

The disadvantages of the UWB system are that it is difficult to achieve long-distance transmission in the case of limited power, and that it is easy to produce inter-code crosstalk, which reduces the signal transmission efficiency and quality of the UWB system. In order to solve these problems, multiple-input multiple-output (MIMO) technology was introduced, which can increase the channel capacity and improve the transmission quality by means of using multiple antenna elements in the transmitter and receiver [12]. The combination of MIMO and UWB technologies can increase the signal transmission distance without extra energy consumption and decrease the disadvantage caused by multipath fading. In many practical scenarios, MIMO antennas in UWB application systems are required to be as small as possible to allow integration of the systems. However, a strong coupling might be generated between antenna elements within too-small distances. It is necessary for us to increase the isolation between them. To decrease coupling, some methods have been introduced, such as conductor branches [13], etching slots [14], loading neutralization lines [15,16], and other reasonable layouts of antenna elements [17,18,19,20,21,22].

In [2], a UWB dual-polarization antenna with high polarization isolation for a passive radar system is investigated. The antenna is composed of a modified Vivaldi antenna and a folded dipole log periodic antenna. However, the working bandwidth of the antenna is narrow and cannot cover the UWB band, and the antenna’s structure is complex. In [8], a three-notched UWB antenna is designed. A modified maple-leaf-shaped main radiating element with partial ground is used in this design. However, the design is not combined with MIMO technology. In [12], a UWB MIMO antenna with a reasonably compact size is presented. The antenna contains two radiating components, each of which is made up of three elliptically shaped patches situated 60 degrees apart. However, the notch structure is not designed to solve the interference of narrowband wireless communication bands with the UWB system, and no time domain analysis of the antenna is shown. In [23], a bio-inspired leaf-shaped antenna is presented for wideband and sensing applications; however, it has a large dimensional size of 314 × 121 mm^2^. The design is not suitable for handheld devices. In [24], a UWB MIMO antenna using a black carbon film reduces the coupling effect due to absorption loss, but the system cost of adopting this approach increases.

In this approach, we use a UWB antenna, but the interferences of the WiMAX and ITU bands must be filtered. Therefore, a UWB antenna with two-notched characteristics is investigated. A U-shaped slot was etched on the radiation patch to get the WiMAX band notch, and an inverted U-shaped slot was etched on the feeder line to get the ITU band notch. Adjusting its size, we obtained a bandwidth from 2.1 to 14.55 GHz of the antenna with stopbands of 3.28–3.75 GHz and 7.98–8.58 GHz. This design meets the requirements for the IEEE 802.16 standard. To adapt the system for higher-quality transmission, a combination of UWB technology and MIMO antenna technology is also studied. So, to use the designed antenna element, we consider the MIMO application. The two-element UWB antenna mentioned above is put in parallel style. Two rectangular branches (RBs) are added between them, which can achieve 17 dB of isolation. The notched bands were not affected. This style is only used in the one-input one-output case. In order to adapt it to real MIMO application scenarios, a four-element UWB antenna is also designed and is put into orthogonal style. Then, a cross-shaped rectangular patch is used as an isolation component. The isolation can reach 20 dB between each antenna element. The notched bands, WiMAX and ITU, were not changed. The simulation and measurement results show that the two proposed MIMO antennas have good performance while being compact in size. They can be widely used in various wireless communication systems such as automotive applications, wireless diversity applications, and portable mobile devices.

The rest of the content is organized as follows. A two-element and a more compact four-element orthogonal MIMO antenna are proposed with two-notched characteristics at the WiMAX and ITU bands. The total antenna structure design with its geometric specifications is introduced in Section 2. The notch and decoupling design of the two antennas are simulated and the corresponding parameters and principles are analyzed in Section 3, where the scattering matrix, radiation pattern, and the current distribution are simulated. Fabricated samples are measured in Section 4, and results from the simulation and experiment are discussed. Section 5 presents the time domain analysis of the UWB antenna element. Finally, conclusions are drawn in Section 6.

## 2. Antenna Structure Design

**Figure 1:**
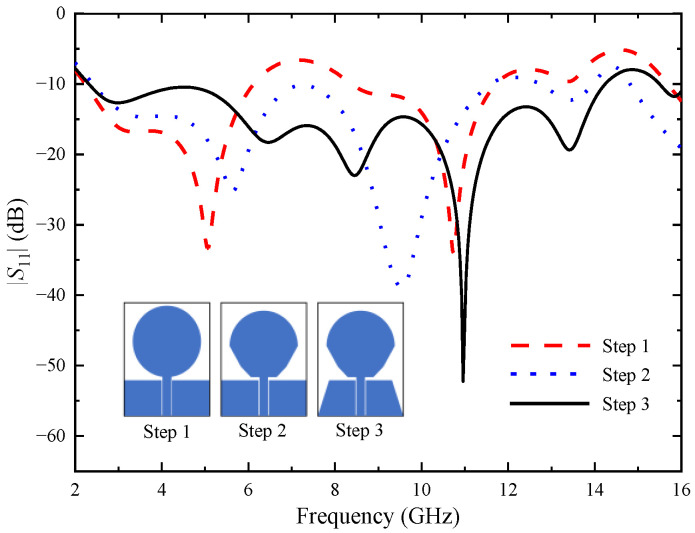
Evolution process of a single antenna element.

A single UWB antenna element design is based on a circular monopole antenna and the structuring of the antenna is conducted in three steps. The monopole antenna has the advantages of simple structure, easy installation and operation, small size, light weight and low cost. Figure 1 exhibits the antenna evolution. For “Step 1”, it can be seen from the simulation reflection coefficients that the antenna operates in the frequency bands from 2.24–6.12 GHz and 8.39–11.71 GHz, which cannot cover the desired frequency range. To extend the antenna working bandwidth, replace the lower half of the circular patch with two inverted trapezoid patches, as shown in “Step 2”. The working bandwidth changes to 2.41–11.59 GHz and 12.73–13.96 GHz. However, there is still a stopband between the two passband bands, and two right triangles are cut at the edge of the ground plane, as shown in “Step 3”. The working bandwidth of the antenna is changed to 2.31–14.55 GHz, which not only covers the UWB band, but also covers the X-band. Our investigation will be built around this UWB antenna element.

**Figure 2:**
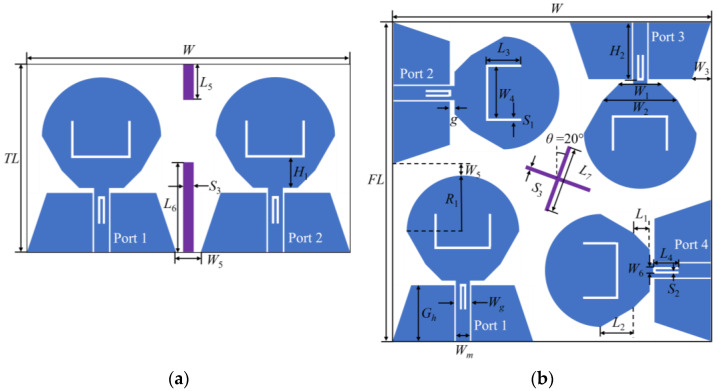
Schematic diagram of the UWB MIMO antenna structure: (**a**) two-element MIMO antenna, two rectangular branches (in purple bar) added into the middle to implement the isolation between two elements; (**b**) four-element MIMO antenna, cross-shaped branch (in purple line) added into the middle to implement the isolation between four elements.

One MIMO antenna consists of two elements and the other of four elements, as shown in Figure 2a and Figure 2b, respectively. The two MIMO antennas have the same antenna element and each element is fed by the coplanar waveguide (CPW). Two U-shaped slots are etched on the antenna to filter the interferences of the WiMAX and ITU bands. The width of the feeder is *W_m_* to meet the impedance matching of 50 Ω. In this paper, U-shaped slots are etched on the radiation patch of the antenna element to realize the notch of WiMAX band, and inverted U-shaped slots are etched on the feeder of the antenna element to obtain the notch of ITU band. To obtain the notch property, the most important thing to calculate is the total length of the etched slot. The total length of the slot can be estimated using Formulas (1) and (2):

(1) $$f_{n}=\frac{c}{2L\sqrt{\varepsilon_{e}}}$$

(2) $$\varepsilon_{e}=\frac{\varepsilon_{r}+1}{2}$$

where *f_n_* is the center frequency of notch; *c* is the speed of light; *L* is the length of the slot; *ε_e_* is the equivalent permittivity; *ε_r_* is the dielectric constant of the dielectric substrate. According to Formulas (1) and (2), the length of the U-shaped slot is about 26 mm when the center frequency is 3.5 GHz, and the length of the inverted U-shaped slot is about 11 mm when the center frequency is 8.3 GHz. After simulating and optimizing with the electromagnetic simulation software High Frequency Structure Simulator (HFSS), it is found that when the length of the U-shaped slot is 25.8 mm and the length of the inverted U-shaped slot is 11.8 mm, the stopband characteristics are the best, which can completely cover the WiMAX and ITU bands, respectively.

The antenna elements of the two-element MIMO antenna are placed symmetrically, and two RBs are loaded between the antenna elements. The two RBs can generate two new current paths, reduce the coupling current between the ports, and improve the isolation between the antenna elements. The four antenna elements of the four-element MIMO antenna are placed orthogonal to each other, and the antenna elements have different polarization modes, resulting in a mismatch of polarization between adjacent antenna elements, thus improving the isolation degree between antenna elements. In order to further improve the isolation degree of the four-element MIMO antenna, a cross-shaped branch is added in the center of the dielectric substrate. The geometric dimensions of the two MIMO antennas are shown in Table 1.

## 3. Simulation Result Analysis

### 3.1. Notch Design and Parameter Analysis

**Figure 3:**
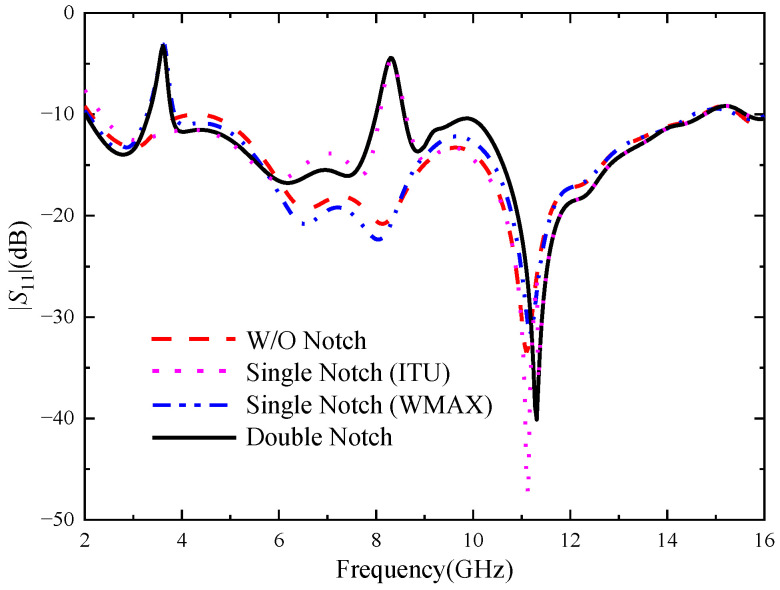
Reflection coefficients of MIMO antenna elements with different numbers of notches.

In order to avoid the interference problem of the narrowband communication system, U-shaped slots in the radiation patch of the UWB antenna and inverted U-shaped slots in the feeder are used to generate notch properties. In order to verify the mutual independence of the two notch structures, Figure 3 shows the |*S*|-parameter simulation curve corresponding to the UWB MIMO antenna when no notch structures and different numbers of notch structures are introduced, respectively.

The non-notch curve in the figure represents the reflection coefficient of the UWB MIMO antenna without a notch structure. It can be seen that the working bandwidth of the antenna is 2.12–14.73 GHz, which can cover the UWB band. When only the U-shaped slot is loaded, the working bandwidth of the antenna changes to 2.05–14.54 GHz, and the notch appears in the band of 3.28–3.75 GHz, which just covers the WiMAX band. When only the inverted U-shaped slot is loaded, the working bandwidth changes to 2.25–14.78 GHz, and the notch appears in the band of 8.01–8.61 GHz, which just covers the ITU band. When the two slots are etched on the antenna, the working bandwidth changes to 2.02–14.74 GHz, and the notch are generated in the 3.28–3.75 GHz and 7.99–8.58 GHz bands, respectively. The change in the parameters of each slot only has an effect on the corresponding notch band, and has little effect on the other parameters of the antenna and the notch band, so the two notch structures can play a relatively independent role.

**Figure 4:**
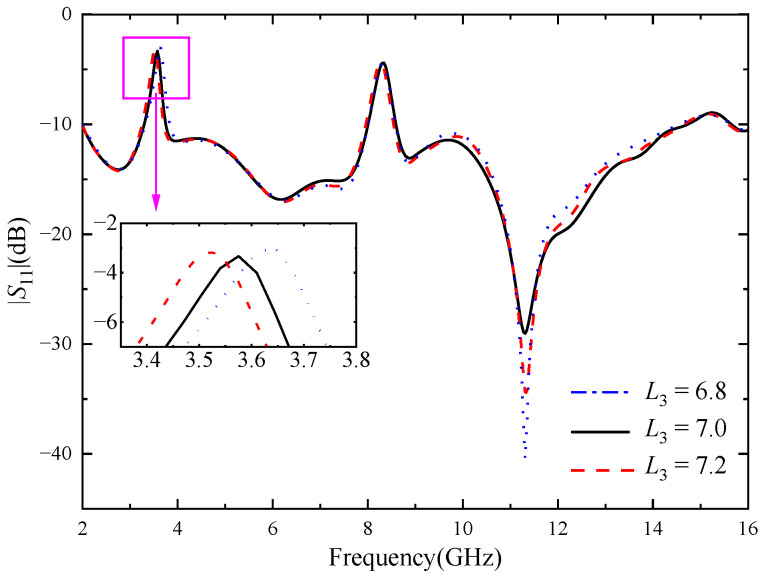
Reflection coefficient of different *L*_3_, the unit of *L*_3_ size (mm).

**Figure 5:**
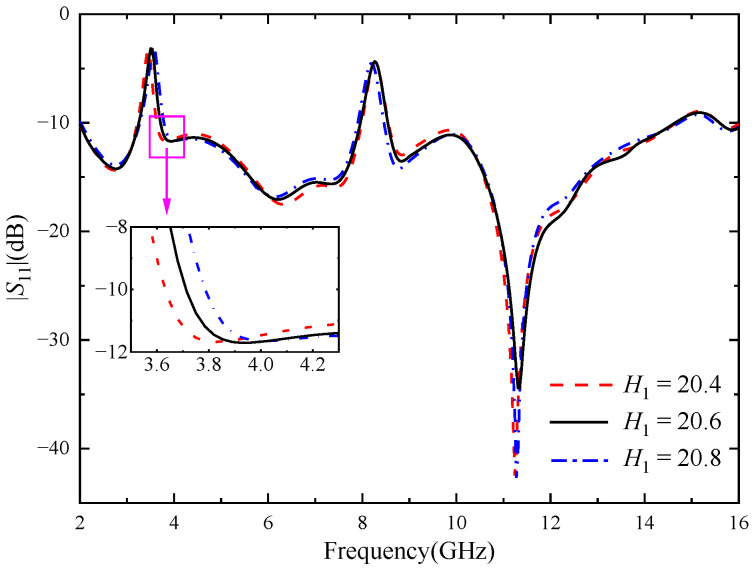
Reflection coefficient of different *H*_1_, the unit of *H*_1_ size (mm).

In order to study the influence of U-shaped slot and inverted U-shaped slot on the notch characteristics, the key dimensions of the two kinds of notch structures were parameterized and simulated. As shown in Figure 4, when *L*_3_ increases from 6.8 mm to 7.2 mm and other parameters remain unchanged, the center frequency of the notch band 3.28–3.75 GHz changes significantly, decreasing from 3.6 GHz to 3.5 GHz. When *L*_3_ = 7.0 mm, the stopband bandwidth of the antenna just covers the WiMAX band. Figure 5 shows the influence of the location of the U-shaped slot on antenna. When *H*_1_ increases from 20.4 mm to 20.8 mm, the center frequency of the 3.28–3.75 GHz notch band does not change significantly, but the stopband bandwidth gradually increases. It can be seen from the above analysis that the notch band can be controlled flexibly through adjusting the length and position of the slot.

**Figure 6:**
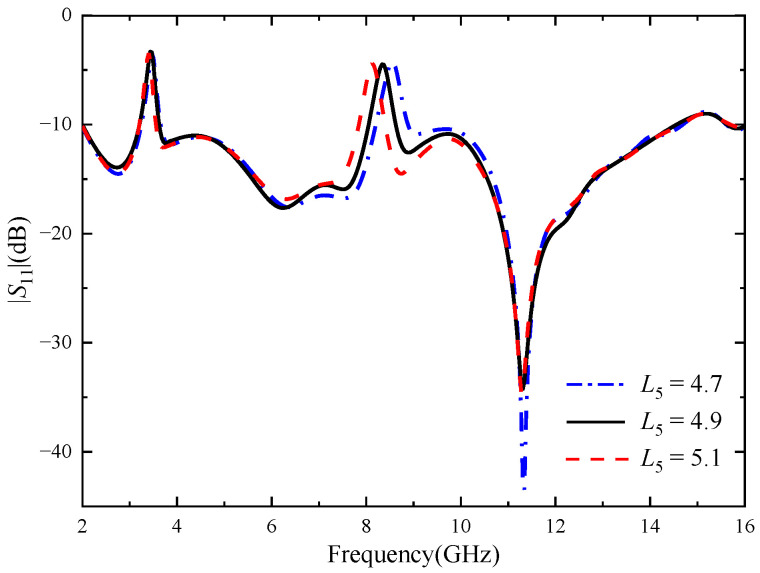
Reflection coefficient of different *L*_4_, the unit of *L*_4_ size (mm).

Figure 6 shows the reflection coefficient for different values of the key parameter *L*_4_ of the inverted U-shaped slot. As shown in Figure 5, when other parameters remain unchanged and *L*_4_ increases from 4.7 mm to 5.1 mm, the notch band 3.28–3.75 GHz does not change significantly, and the center frequency gradually decreases. Through optimizing the key parameters of the two notch structures, it can be seen that the U-shaped slot mainly affects the notch band at 3.28–3.75 GHz, and the inverted U-shaped slot mainly affects the notch band at 7.99–8.58 GHz, with little mutual interference.

**Figure 7:**
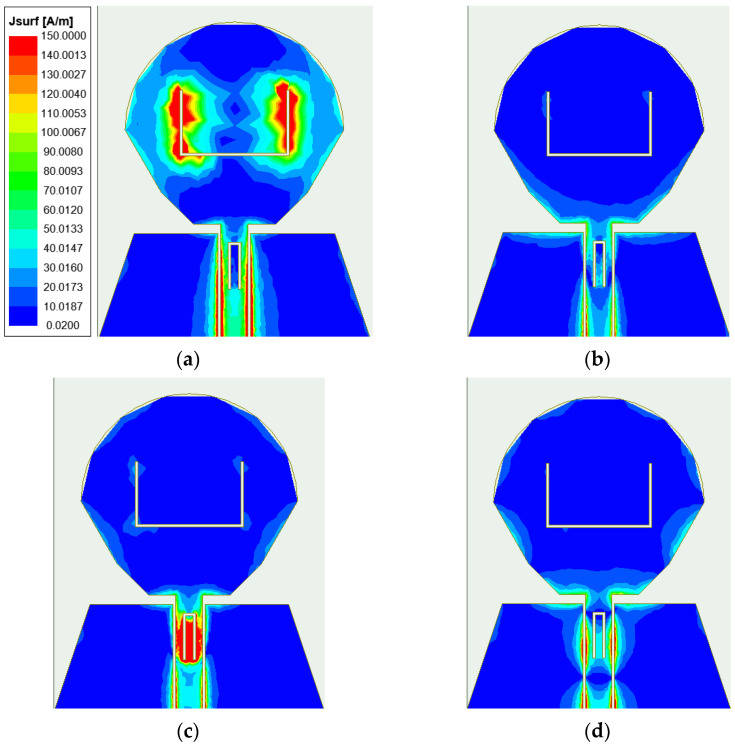
Antenna surface current distribution at four frequency points: (**a**) 3.55 GHz; (**b**) 5.5 GHz; (**c**) 8.25 GHz; (**d**) 12.5 GHz.

In order to understand the principle of antenna notches, the antenna was further analyzed. Taking the element antenna as an example, the surface current distribution was simulated and analyzed. Figure 7 shows the antenna surface current distribution at two notch center frequencies of 3.55 and 8.25 GHz and two passband frequency points of 5.5 and 12.5 GHz. It can be seen from the figure that the current at 3.55 GHz is mainly concentrated in the U-shaped slot, and the current at 8.25 GHz is mainly concentrated in the inverted U-shaped slot. It can be seen from Figure 7a,c that the energy is mainly concentrated at the two notch structures, while Figure 3 shows that the reflection coefficient at the two frequency points of 3.55 and 8.25 GHz is higher than −10 dB, which cannot achieve good impedance matching, and it can be proved that the energy here cannot effectively radiate outward, so the notch function is realized. At the passband frequencies of 5.5 GHz and 12.5 GHz, the electric field on the patch and ground plane is evenly distributed, and the electric field near the feeder and the power port is the largest, and the energy can be radiated out, which confirms that the antenna can work normally on the passband frequency.

### 3.2. Decoupling Design and Parameter Analysis

#### 3.2.1. Two-Element MIMO Antenna

In order to study the influence of RBs on MIMO antennas and the decoupling effect, two-element MIMO antennas without and with RBs are simulated and analyzed, and the parameters of RBs are scanned and analyzed.

**Figure 8:**
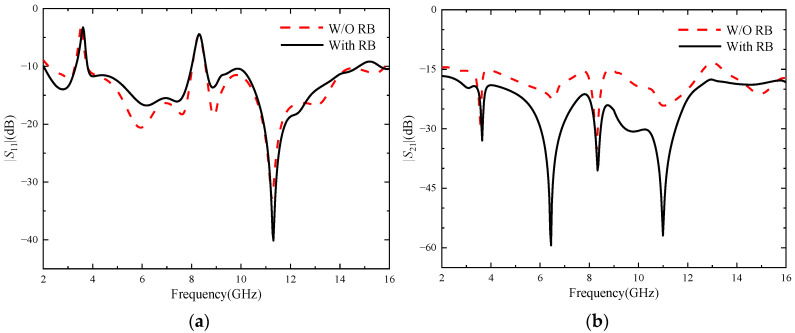
The |*S*|-parameters with or without RB: (**a**) |*S*_11_|; (**b**) |*S*_21_|.

Figure 8 shows the comparison of |*S*|-parameters before and after antenna loading RBs: (a) represents |*S*_11_|, (b) represents |*S*_21_|. As can be seen from Figure 8a, loading RB will deteriorate the antenna’s impedance matching in the 4–10 GHz frequency band, but the influence is small and the antenna can still meet the performance indexes of UWB antennas. As can be seen from Figure 8b, when the RBs are not loaded, the antenna’s isolation degree is lower than 15 dB in the 8.7–8.9 GHz and 12.64–13.41 GHz bands, and the isolation effect is poor, which does not meet the basic requirements for designing MIMO antennas. However, after loading the RBs, the antenna isolation is obviously improved, especially in the 5–13 GHz frequency band, the antenna isolation is higher than 20 dB, and the antenna isolation is also higher than 17 dB in the other frequency bands of the working frequency band, with an average increase of about 7 dB, indicating an obvious isolation effect. In short, figures (a) and (b) show that RBs effectively inhibit electromagnetic coupling between antenna elements and significantly improve the isolation degree between elements. At the same time, the |*S*_11_| of the antenna changed little and remained below −10 dB in the UWB band, and the antenna did not exhibit an impedance mismatch phenomenon.

In order to further study the influence of isolation structure on antenna performance, the key dimensions of isolation structure are simulated and optimized. Port 1 is set as the excitation port.

**Figure 9:**
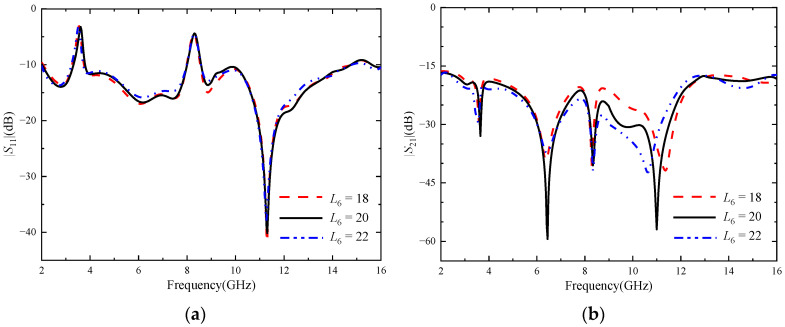
|*S*|-parameters with different *L*_6_, the unit of *L*_6_ size (mm): (**a**) |*S*_11_|; (**b**) |*S*_21_|.

Figure 9 represents antenna |*S*|-parameters corresponding to different *L*_6_ values, where (a) represents |*S*_11_| and (b) represents |*S*_21_|. As can be seen from Figure (b), with the increase of *L*_6_, the degree of isolation between the two antenna elements also increases, resulting in a better decoupling effect. As can be seen from figure (a), the change of *L*_6_ has little effect on the overall antenna impedance matching, but it has some effect on the range of the notch band. When *L*_6_ = 20 mm, the notch band just covers the WiMAX band. In summary, when *L*_6_ = 20 mm, the antenna can not only have high isolation, but also accurately cover the WiMAX band, and its isolation is higher than 15 dB, which meets the basic design requirements of a MIMO antenna and has a good decoupling effect.

Through simulation and optimization, it is found that other dimensions of isolation branches have little influence on antenna impedance matching, and have no significant effect on the improvement of isolation degree, so it will not be analyzed and described.

**Figure 10:**
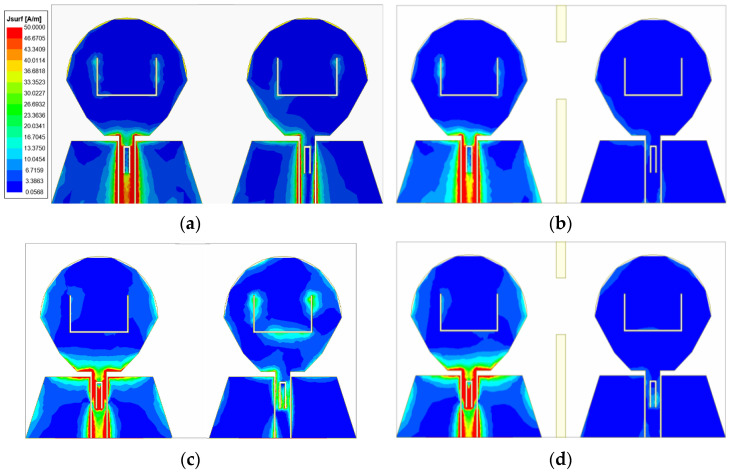

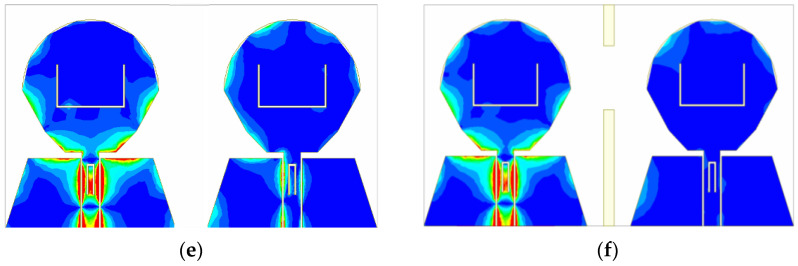
Antenna surface current distribution at (**a**) 3 GHz without adding RB; (**b**) 3 GHz with adding RB; (**c**) 7.25 GHz without adding RB; (**d**) 7.25 GHz with adding RB; (**e**) 11.6 GHz without adding RB; (**f**) 11.6 GHz with adding RB.

In order to more directly reflect the function of RBs, Figure 10 shows the surface current comparison diagram of without and with RBs at frequencies of 3 GHz, 7.25 GHz and 11.6 GHz. It can be clearly seen from the comparison figure that when the RB is not added, there are more green areas on the feeder and radiation patch of the antenna element where port 2 is located, which indicates that the surface current intensity of the antenna element where port 2 is located is relatively large, and further proves that part of the energy of port 1 is coupled to port 2. When the rectangular decoupling structure was added between the two antenna elements, it could be seen that the surface current intensity of the radiation patch of the antenna element where port 2 was located decreased significantly, which indicated that most of the energy was separated by the RB, and also proved that port 1 had little energy coupled to port 2. The comparison shows that the decoupling structure can isolate the interference between antenna elements well, improving the isolation degree between two ports.

#### 3.2.2. Four-Element MIMO Antenna

**Figure 11:**
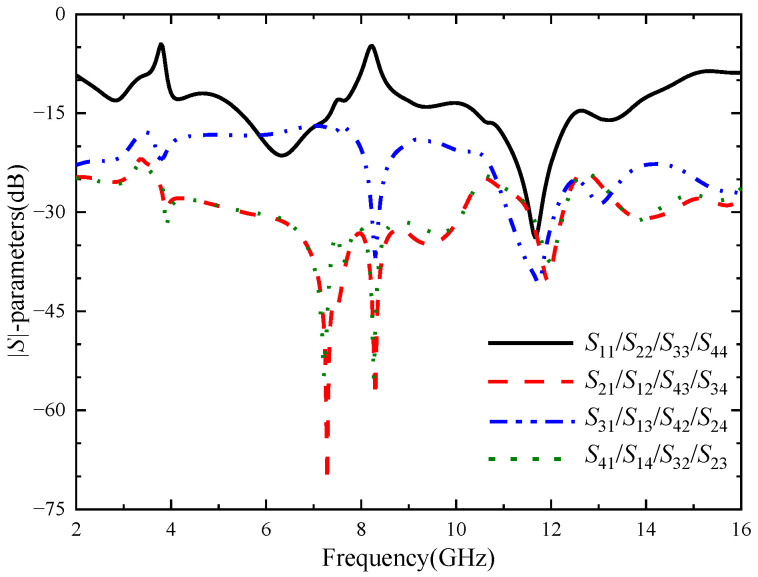
|*S*|-parameters of the four-element MIMO antenna.

Increasing the number of MIMO antenna elements will increase the difficulty of decoupling. Therefore, each element of the four-element MIMO antenna is placed orthogonally first. The orthogonal placement of antenna elements will lead to the polarization mismatch of adjacent antennas, thus improving the isolation degree between antenna elements. Due to the four-element MIMO antenna having a symmetrical structure, the |*S*_11_| of each antenna element is equal, while the isolation degree between antenna elements meet |*S_ij_*| = |*S_ji_*| (*i* ≠ *j*; *i*, *j* ≤ 4). In order to more conveniently study the variation of the |*S*|-parameters of this MIMO antenna, only the |*S*|-parameters of port 1 can be studied. Figure 11 shows the |*S*|-parameters of the four-element MIMO antenna. It can be seen from the figure that the working bandwidth of the antenna is 2.17–14.71 GHz, in which the notch band of the U-shaped slot is 3.26–3.79 GHz, and the notch band of the inverted U-shaped slot is 7.93–8.51 GHz. When port 1 is excited, the isolation degree between port 1 and port 2 and between port 1 and port 4 is higher than 23 dB, and the isolation degree between port 1 and port 3 is higher than 15 dB in the working bandwidth. It can be proved that the orthogonal placement of antenna elements can reduce the coupling between antenna elements and obtain a good polarization diversity effect. Since the isolation between port 1 and port 3 just meets the basic design requirements, considering that the addition of antenna elements will increase the instability of antenna operation, it is necessary to further improve the isolation between port 1 and port 3.

**Figure 12:**
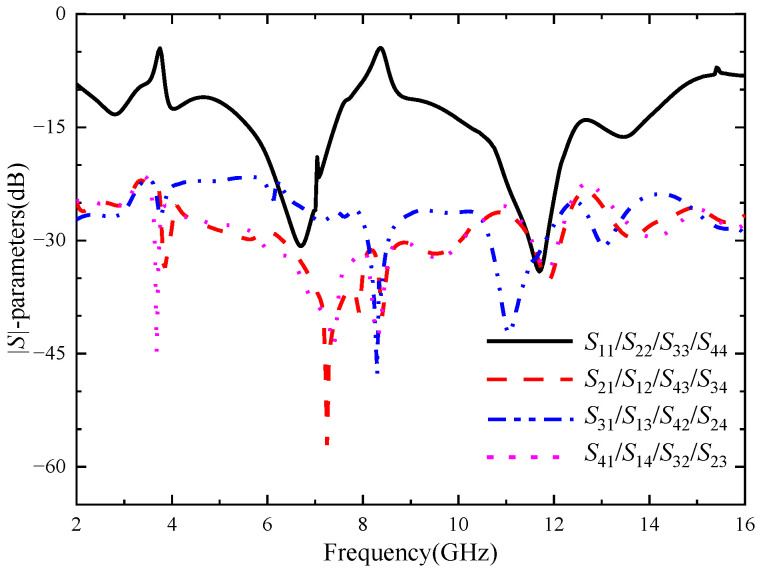
The |*S*|-parameters of the four-element MIMO antenna with the cross branch.

In order to further improve the isolation degree between the antenna elements of the four-element MIMO antenna, the center of the dielectric substrate is loaded with a cross-shaped branch for decoupling. In order to study the influence of cross-shaped branch on antenna performance, the key parameters of the cross-shaped branch are scanned and optimized. Figure 12 is the |*S*|-parameters of the four-element MIMO antenna after loading the cross-shaped branch. As can be seen from Figure 12, the addition of the cross-shaped branch significantly improves the isolation degree between port 1 and port 3, but has little impact between port 1 and other ports. The isolation degree between ports is higher than 21 dB, which has a good decoupling effect. The working bandwidth of the four-element MIMO antenna is 2.13–14.62 GHz, in which the notch band of the U-shaped slot becomes 3.27–3.77 GHz, and the notch band of the inverted U-shaped slot becomes 7.89–8.65 GHz. The two frequency bands can still cover the WiMAX and ITU band, respectively. The antenna has accurate notch property and good impedance matching.

**Figure 13:**
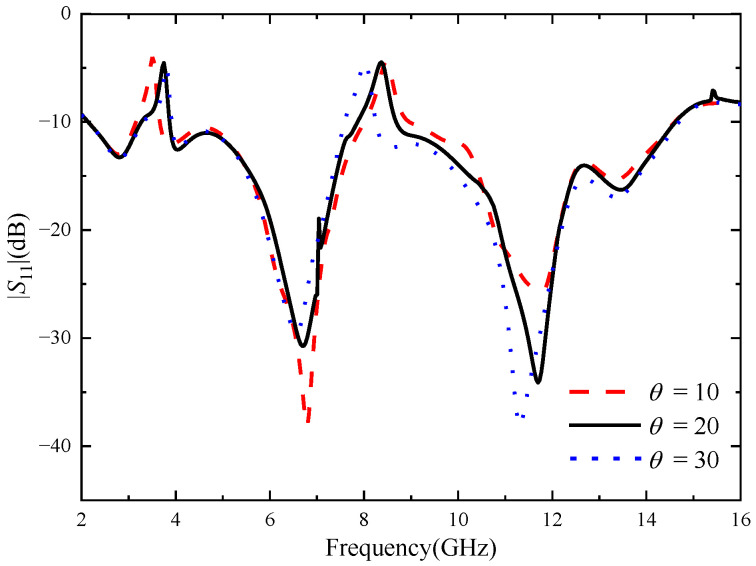
|*S*_11_| with different *θ*, the unit of *θ* size (degree).

**Figure 14:**
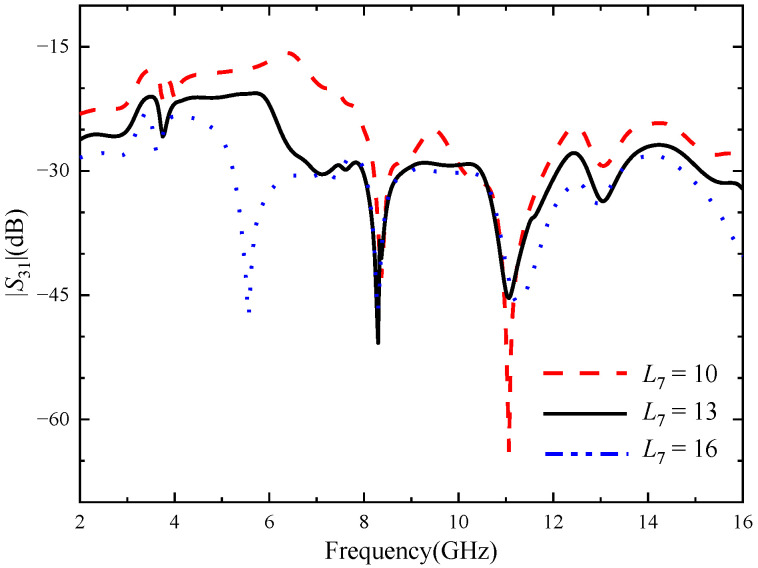
|*S*_31_| with different *L*_7_, the unit of *L*_7_ size (mm).

Through simulation analysis, it is found that the rotation angle *θ* of the cross-shaped branch has a great influence on the notch band of the four-element MIMO antenna, and the length of the cross branch has a significant effect on improving the isolation between port 1 and port 3. The *θ* was set as 10–30 deg, and the step size was set as 2 deg. Figure 13 shows |*S*_11_| with different *θ* values. It can be seen from the figure that when *θ* increases from 10 to 30 deg, the center frequency of the notch band corresponding to the U-shaped slot gradually increases, while that corresponding to the notch band corresponding to the inverted U-shaped slot gradually decreases. When *θ* = 20 deg, the two notch bands exactly cover the WiMAX and ITU band. The *L*_7_ is set from 10 to 16 mm, and the step is set to 1 mm. |*S*_31_| corresponding to different values of *L*_7_ is shown in Figure 14. As can be seen from the figure, as *L*_7_ increases, the isolation between port 1 and port 3 gradually increases. However, with the change in the value of *L*_7_, the accuracy of the notch band will be slightly affected. When *L*_7_ = 13 mm, the antenna has a high isolation degree and the notch band can achieve accurate coverage.

**Figure 15:**
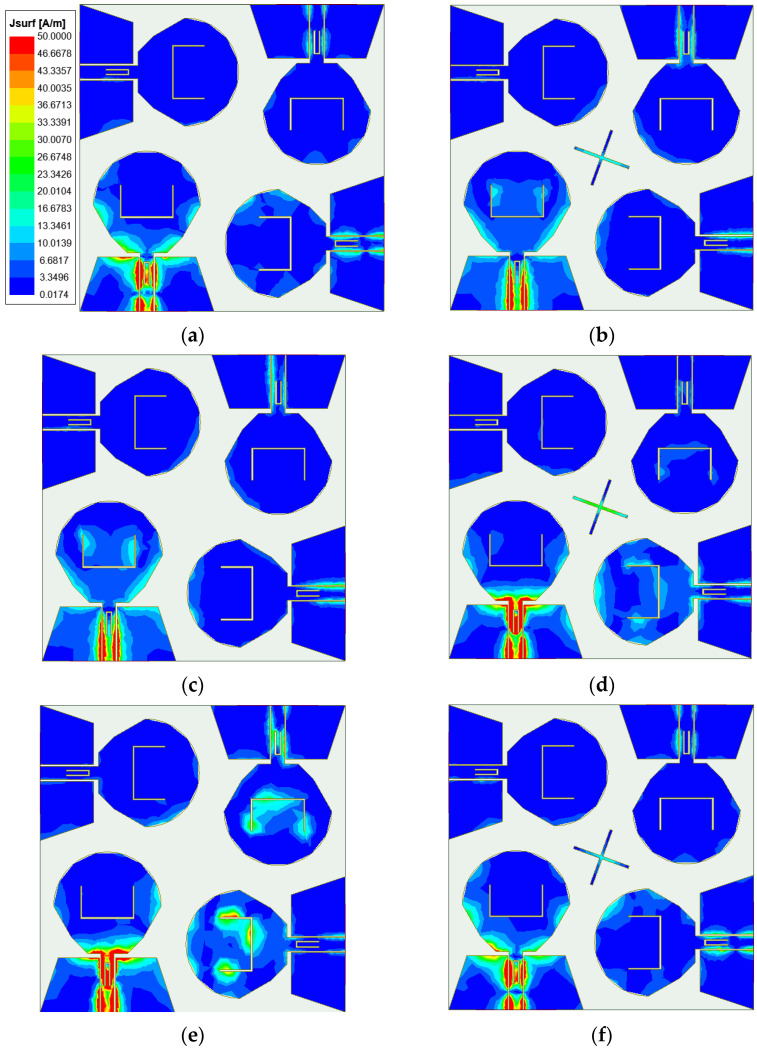
Antenna surface current distribution at (**a**) 4.5 GHz without adding cross-shaped branch; (**b**) 4.5 GHz with adding cross-shaped branch; (**c**) 7.25 GHz without adding cross-shaped branch; (**d**) 7.25 GHz with adding cross-shaped branch; (**e**) 11.6 GHz without adding cross-shaped branch; (**f**) 11.6 GHz with adding cross-shaped branch.

In order to more directly reflect the effect of orthogonal placement of antenna elements and the isolation effect after loading the cross-shaped branch, Figure 15 shows the surface current comparison diagram of the four-element MIMO antenna at the frequencies of 4.5 GHz, 7.25 GHz, and 11.6 GHz. As can be seen from Figure 15, when the cross-shaped branch is not loaded, part of the energy of port 1 is coupled to other ports. From the current distribution on the antenna surface, the coupled energy is small, which proves that the isolation degree can be improved through placing antenna elements orthogonal to each other. Through observing the current distribution of the antenna surface with the cross-shaped branch loaded, it is found that the energy coupled from port 1 to other ports is less than that without the cross-shaped branch loaded. The currents on both sides of the cross-shaped branch form opposite directions, and the electric fields radiated by the cross-shaped branch cancel each other in opposite directions to realize the decoupling effect, which further proves that the cross-shaped branch can improve the isolation between antenna elements.

**Figure 16:**
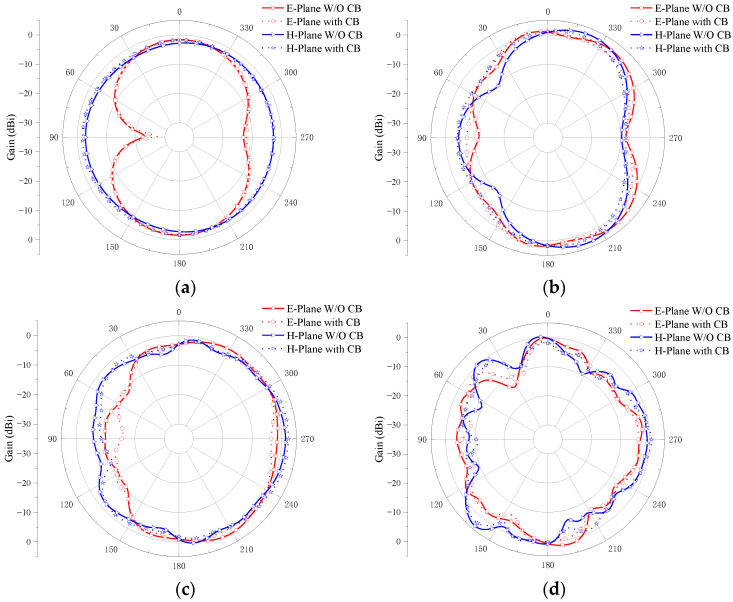
Four-element MIMO antenna simulation radiation pattern with and without adding cross-shaped branch (CB) at (**a**) 2.8 GHz; (**b**) 6.5 GHz; (**c**) 10.6 GHz; (**d**) 14.3 GHz.

Figure 16 illustrates the simulated radiation pattern of the four-element MIMO antenna before and after adding the cross-shaped branch at the operating frequencies of 2.8, 6.5, 10.6, and 14.3 GHz. It can be seen from Figure 16 that there is little difference in the radiation pattern of the antenna before and after adding the cross-shaped branch. It is proved that the radiation performance of the antenna is less affected after adding the cross-shaped branch.

## 4. Measured Results and Analysis

**Figure 17:**
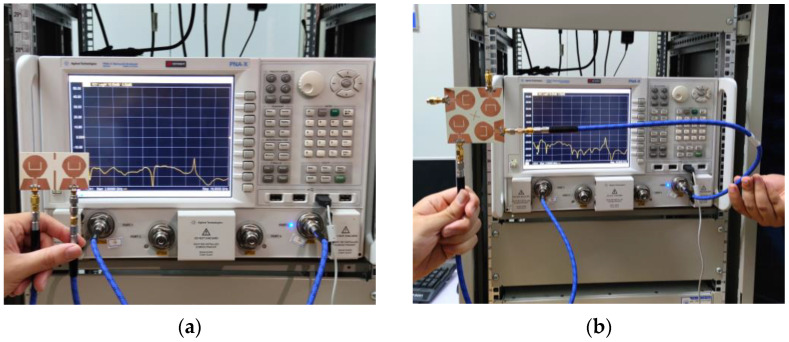
Photographs of the measured |*S*|-parameter scenarios using vector network analyzer Keysight N5244A: (**a**) two-element MIMO antenna; (**b**) four-element MIMO antenna.

**Figure 18:**
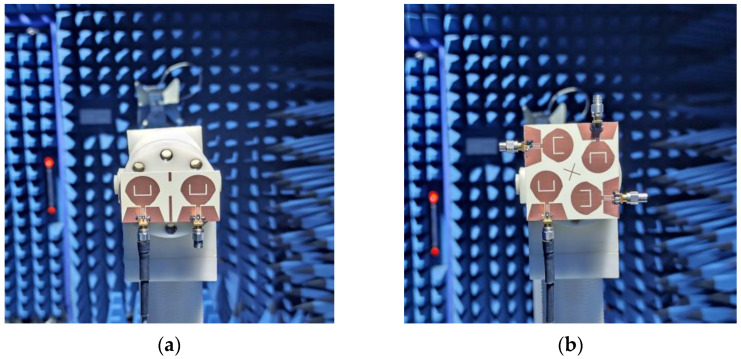
Photographs of far-field radiation pattern measurements using a microwave anechoic chamber: (**a**) two-element MIMO antenna; (**b**) four-element MIMO antenna.

In order to verify the performance of the two MIMO antennas designed, the antennas are manufactured according to the data in Table 1. Both MIMO antennas are fed via coplanar waveguide (CPW) and printed on F4BTM440 dielectric substrates with a relative dielectric constant of 4.4 and a tangent loss of 0.0015. A vector network analyzer (N5244A) and microwave anechoic chamber were used to measure the MIMO antenna |*S*|-parameter and far-field radiation pattern, as shown in Figure 17 and Figure 18.

### 4.1. S-Parameter

#### 4.1.1. Two-Element MIMO Antenna

**Figure 19:**
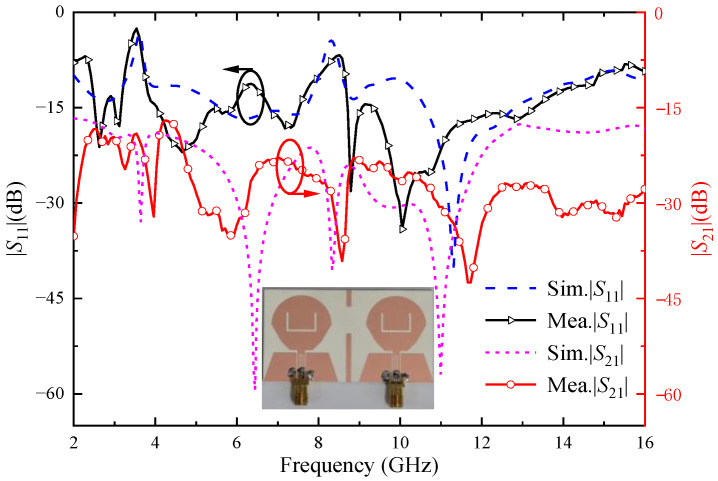
Simulation and measured |*S*|-parameters of the proposed two-element MIMO antenna; black circle with arrow means pointing to *S*_11_; red circle with arrow means pointing to *S*_21_.

Figure 19 shows the two-element UWB MIMO antenna simulation and the measured |*S*|-parameters, due to the symmetry of the antenna unit, only consider the |*S*|-parameters of port 1. As can be seen from the figure, the measured working bandwidth of the antenna is 2.45–14.88 GHz, which can cover the UWB band. The notch band corresponding to the U-shaped slot is 3.26–3.75 GHz, and the notch band corresponding to the inverted U-shaped slot is 7.96–8.65 GHz, which can cover the WiMAX and ITU band, respectively. The measured isolation between two antenna ports is higher than 18 dB. Through comparing the simulation results and the measured results in Figure 19, it can be seen that the measured |*S|*-parameters are roughly consistent with the simulated |*S*|-parameters. However, due to the errors caused by antenna machining, the welding process, and network analysis instruments, the measured and simulated values have a small difference.

#### 4.1.2. Four-Element MIMO Antenna

**Figure 20:**
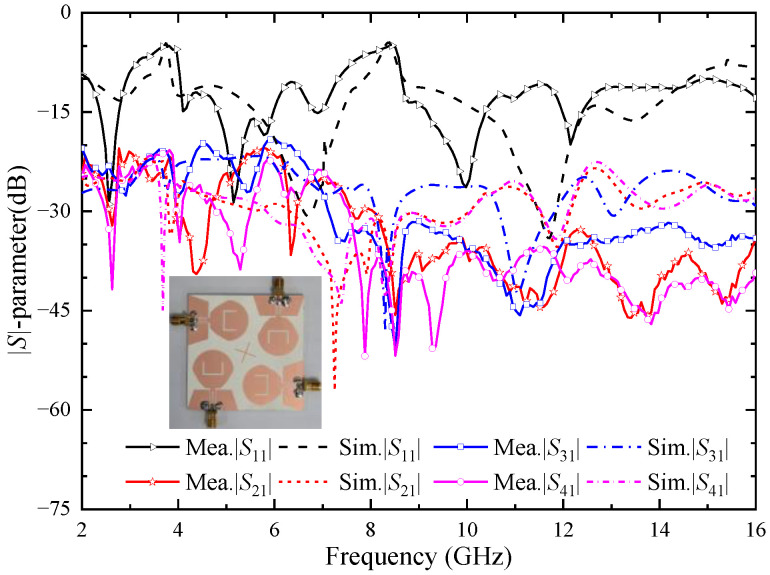
Simulation and measured |*S*|-parameters of the proposed four-element MIMO antenna.

Figure 20 shows the simulation and the measured |*S*|-parameters of the four-element UWB MIMO antenna; due to the symmetry of the antenna element, only consider the |*S*|-parameters of port 1. As can be seen from the figure, the measured working bandwidth of the antenna is 2.14–14.95 GHz, which can cover the UWB band. The notch band corresponding to the U-shaped slot is 3.02–3.99 GHz, and the notch band corresponding to the inverted U-shaped slot is 7.56–8.58 GHz. The bandwidth of the two notch bands becomes wider. However, WiMAX and ITU bands can still be covered separately. The measured isolation degree between each antenna port is higher than 20 dB, which proves that the isolation degree between antenna elements is high and they can maintain independent normal operation. Through comparing the simulation results and the measured results in Figure 18, it can be seen that the measured |*S|*-parameters are roughly consistent with the simulated |*S*|-parameters, which proves that the antenna has good performance.

### 4.2. Radiation Pattern

#### 4.2.1. Two-Element MIMO Antenna

**Figure 21:**
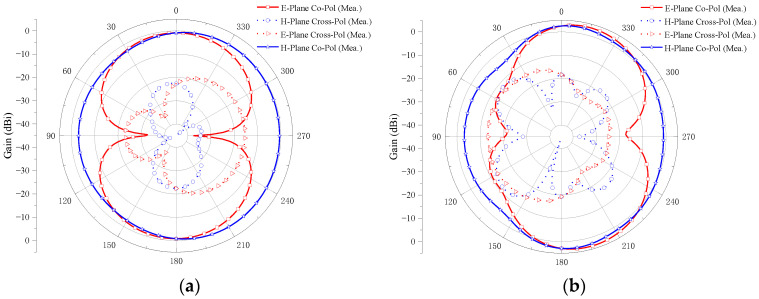

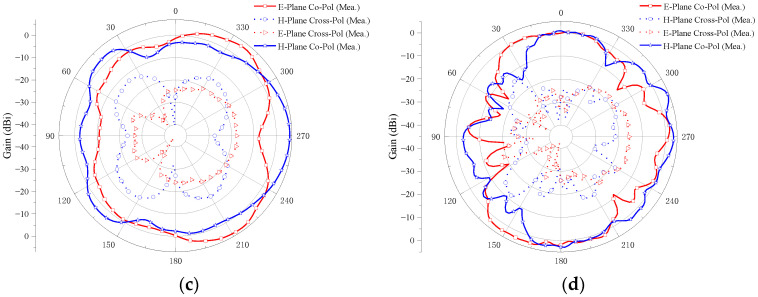
The measured radiation pattern of the proposed two-element MIMO antenna: (**a**) 2.8 GHz; (**b**) 6.5 GHz; (**c**) 10.6 GHz; (**d**) 14.3 GHz.

Figure 21 shows the measured radiation pattern of the two-element MIMO antenna at the operating frequencies of 2.8, 6.5, 10.6, and 14.3 GHz when port 1 is excited and port 2 is connected to a 50 Ω load. 

The E-plane co-polarizations of the four sample frequencies have two main beams, and the cross-polarizations are small. Furthermore, the differences between the co-polarizations and the cross-polarizations are larger than 15 dB, which shows good radiation patterns in the E-plane. It can be also observed in Figure 21 that the co-polarizations in the H-plane are omnidirectional, and the cross-polarizations are all smaller for the four samples’ frequencies. Moreover, the differences between the co-polarizations and the cross-polarizations are larger than 17 dB, which exhibits the stable radiation patterns in the H-plane. It also can be seen from the figure that the radiation pattern of the E-plane and H-Plane is good at 2.8, 6.5, and 10.6 GHz, but becomes irregular at 14.3 GHz. With the increase in frequency, the wavelength of the antenna decreases and is no longer much larger than the size of the antenna. The antenna no longer has the characteristics of an electrically small antenna. Therefore, the radiation pattern of the antenna changes and no longer presents the radiation pattern similar to that of a monopole antenna. Although the shape degradation and deformation of the antenna radiation pattern will occur at a high frequency, the radiation intensity still meets the communication requirements of UWB antenna. It can be seen from the radiation pattern of the antenna that the antenna is an omnidirectional antenna, which is suitable for most scenarios of UWB technology application.

#### 4.2.2. Four-Element MIMO Antenna

**Figure 22:**
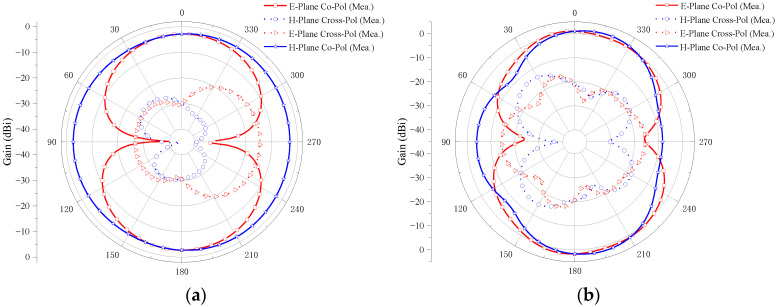

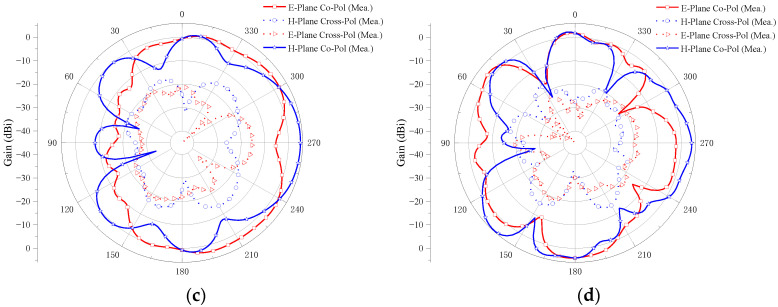
The measured radiation pattern of the proposed four-element MIMO antenna: (**a**) 2.4 GHz; (**b**) 6.9 GHz; (**c**) 10.1 GHz; (**d**) 14.3 GHz.

Figure 22 shows the measured radiation pattern of the four-element MIMO antenna at the operating frequencies of 2.4, 6.9, 10.1, and 14.3 GHz when port 1 is excited and other ports are connected to a 50 Ω load. 

The E-plane cross-polarizations are small. Furthermore, the differences between the co-polarizations and the cross-polarizations are larger than 16 dB, which shows good radiation patterns in the E-plane. It can be also observed from Figure 22 that the co-polarizations in the H-plane are omnidirectional and the cross-polarizations are all smaller for the four sample frequencies. The differences between the co-polarizations and the cross-polarizations are larger than 16 dB, which exhibits the stable radiation patterns in the H-plane. It also can be seen from the figure that the radiation pattern of the E-plane and H-Plane is good at 2.4 and 6.9 GHz, but becomes irregular at 10.1 and 14.3 GHz. Although the antenna radiation pattern is deformed at a high frequency, it still meets the design requirements of a UWB antenna. The antenna is omnidirectional and suitable for most UWB technology application scenarios.

### 4.3. MIMO Diversity Analysis

The envelope correlation coefficient (ECC), diversity gain (DG), total active reflection coefficient (TARC), mean effective gain (MEG), and channel capacity loss (CCL) are important parameters for validating the capability and performance of MIMO antennas.

#### 4.3.1. Envelope Correlation Coefficient (ECC)

The ECC is used to measure the correlation between the channels of MIMO antenna elements. It refers to the correlation between different signal amplitudes received by the antenna. For MIMO antennas, a smaller ECC means weaker channel correlation and better system performance. In general engineering applications, if the ECC is less than 0.5 [25], it can be considered that the antenna channels can work independently. The calculation of the S-parameter for ECC [26] is shown in Formula (3):

(3) $$\rho_{\mathrm{eij}}=\frac{|S_{ii}^{*}S_{ij}+S_{ji}^{*}S_{jj}{|}^{2}}{\left(1-|S_{ii}{|}^{2}-|S_{ji}{|}^{2})(1-|S_{jj}{|}^{2}-|S_{ij}{|}^{2}\right)}$$

**Figure 23:**
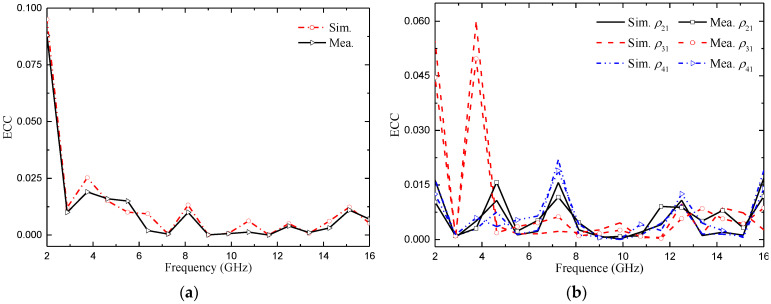
ECC of the proposed antenna: (**a**) two-element MIMO antenna; (**b**) four-element MIMO antenna.

Figure 23 shows the ECC of the two proposed MIMO antennas, which shows that the ECC is very small (<0.02) in the whole passband bandwidth except for the notch band. The ECC values of the two proposed MIMO antennas are low and meet the standard of an ECC of less than 0.5, which indicates that the antennas have good diversity performance and can be effectively applied in multi-antenna systems.

#### 4.3.2. Diversity Gain (DG)

Diversity gain (DG) is another important parameter for measuring the performance of MIMO antenna [26]. The ideal value is 10 dB. It can be calculated as (4):

(4) $$DG=10\sqrt{1-|ECC_{\mathrm{ij}}{|}^{2}}$$

**Figure 24:**
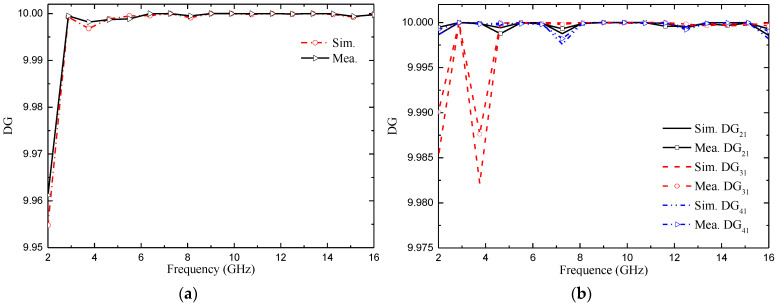
DG of the proposed antenna: (**a**) two-element MIMO antenna; (**b**) four-element MIMO antenna.

The measured and simulated DG of the two proposed MIMO antennas are shown in Figure 24. From Figure 24, we can confirm that the DG of the two MIMO antennas is very close to 10 dB, and the maximum value is 9.98 dB. We also noticed that the value of the diversity gain was very similar for the simulated and measured cases.

#### 4.3.3. Total Active Reflection Coefficient (TARC)

The TARC provides the reflected-incident power ratio, which is related to the coupling between the ports [27]. For an N-port antenna, the TARC ratio can be calculated using Equations (5) and (6):

(5) $$TARC=\frac{\sqrt{{\sum_{n=1}^{N}\left|\left.b_{n}\right|\right.}^{2}}}{\sqrt{{\sum_{n=1}^{N}\left|\left.a_{n}\right|\right.}^{2}}}$$

(6) $$b_{n}=[S]a_{n}$$

where *b* represents the reflected signal, *a* represents the incident signal, *N* depicts the number of elements in the MIMO system, and *S* denotes the scattering parameter. Through deriving the formula, the TARC of the MIMO system can be calculated using the S-parameter [26]. The TARC from port 1 to other ports at a random phase of 0° can be calculated using Equation (7).

(7) $$TARC=-\sqrt{\frac{{\left(S_{11}+S_{1i}\right)}^{2}+{\left(S_{i1}+S_{ii}\right)}^{2}}{2}}(i=2,3,4)$$

**Figure 25:**
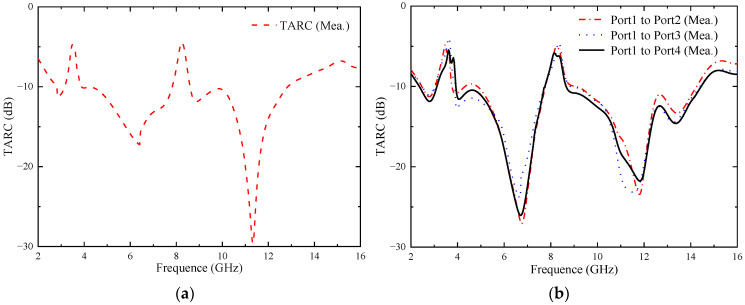
Measured TARC of the proposed antenna: (**a**) two-element MIMO antenna; (**b**) four-element MIMO antenna.

In order for the MlMO antenna to work well, the TARC value must be below −10 dB [22]. Figure 25 represents the measured TARC of the two proposed MIMO antennas. It can be seen from the figure that the TARC values of the two proposed MIMO antennas in the operating frequency band (except the notch band) are lower than −10 dB.

#### 4.3.4. Mean Effective Gain (MEG)

**Figure 26:**
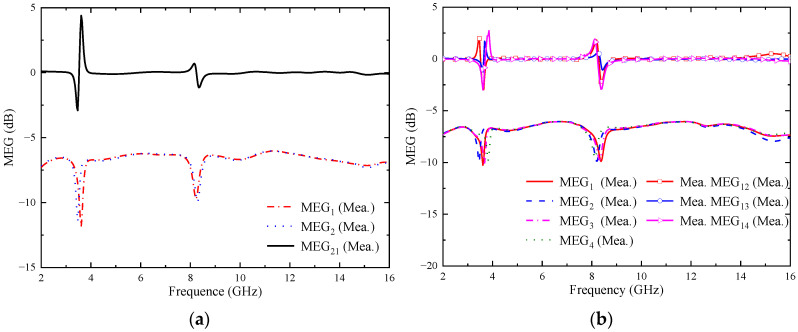
Measured MEG of the proposed antenna: (**a**) two-element MIMO antenna; (**b**) four-element MIMO antenna.

In a multipath fading environment, the MEG can be used to measure the total efficiency of the MIMO system, the total gain, and the effect produced by the propagation environment [28]. It is generally required that the MEG between the elements within the MIMO system is less than 3 dB, and the MEG can be estimated from the S-parameters of each port [29]. *MEG_i_* is calculated using Formula (8). Similarly, *MEG_ij_* is calculated using Formula (9), and the data details are shown in Figure 26.

(8) $$MEG_{i}=0.5\times \left(1-{\sum_{j=1}^{N}\left|\left.S_{ij}\right|\right.}^{2}\right)(i=1,2,3,4)$$

(9) $$MEG_{ij}=MEG_{i}-MEG_{j}(i\neq j)$$

It can be seen that the *MEG_i_* of the two proposed MIMO antennas is lower than 6 dB in the whole operating band, and the *MEG_ij_* is lower than 3 dB. It can be proved that the two proposed MIMO antennas perform well over the whole operating band.

#### 4.3.5. Channel Capacity Loss (CCL)

The correlation between the elements in MIMO channel systems produces a capacity loss [30]. For high data transfer, the value of CCL should be below 0.4 bits/s/Hz [31]. CCL is calculated using Formulas (10)–(12):

(10) $$C_{loss}=-\log_{2}\det(\psi^{R})$$

(11) $$\psi^{R}=\left[\begin{array}{cccc} \psi_{11} & \psi_{12} & \psi_{13} & \psi_{14}\\ \psi_{21} & \psi_{22} & \psi_{23} & \psi_{24}\\ \psi_{31} & \psi_{32} & \psi_{33} & \psi_{34}\\ \psi_{41} & \psi_{42} & \psi_{43} & \psi_{44} \end{array}\right]$$

(12) $$\psi_{ii}=1-\sum_{n=1}^{4}\left(S_{in}^{*}S_{ni}\right);\psi_{ij}=-\sum_{n=1}^{4}\left(S_{in}^{*}S_{nj}\right)$$

**Figure 27:**
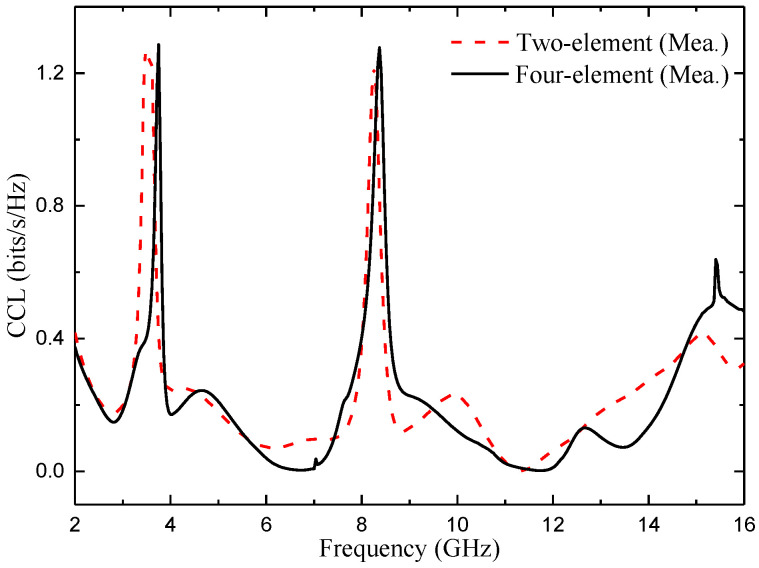
Measured CCL of the two proposed antennas.

The CCL values of the two proposed MIMO antennas are shown in Figure 27. It can be seen from the figure that the CCL value of the two MIMO antennas is lower than 0.4 bit/s/Hz in the whole operating band (except the notch band), which meets the design index.

### 4.4. Radiation Efficiency

As an energy conversion device, an antenna converts high-frequency current energy into electromagnetic wave energy or electromagnetic wave energy into high-frequency current energy. However, due to various losses in the transmission process, such as antenna dielectric loss, copper loss, and component loss, the input antenna power can only be partially converted into electromagnetic wave energy. The efficiency of the antenna is used to characterize the degree of such conversion. The calculation method is shown in Formula (13):

(13) $$\eta =\frac{P_{r}}{P_{in}}=\frac{P_{r}}{P_{r}+P_{d}}$$

where *P_in_* is the power entering the antenna, *P_r_* is the radiated power of the antenna, and *P_d_* is the lost power of the antenna.

**Figure 28:**
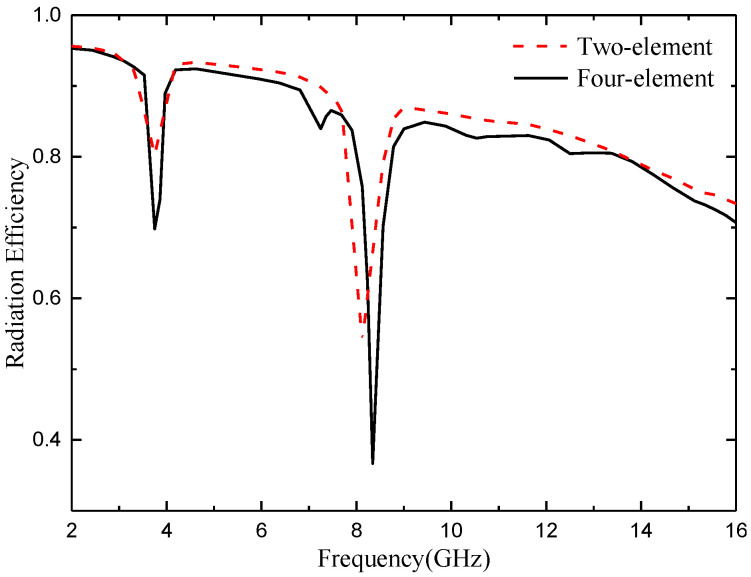
Measured radiation efficiency of the proposed antenna for 2- and 4-element antennas.

The measured radiation efficiency of the two proposed MIMO antennas is shown in Figure 28. It can be seen from the figure that, except for the notch band, the minimum radiation efficiency of the two MIMO antennas is also higher than 80% in the whole passband bandwidth range, which proves that the antennas achieve good radiation energy conversion and fully meet the requirements of wireless devices. However, the radiation efficiency of the antenna decreases obviously in the two notch bands, which further proves that the energy radiation of the antenna in the notch band can be reduced through notch design.

## 5. Time Domain Performance Analysis

**Figure 29:**
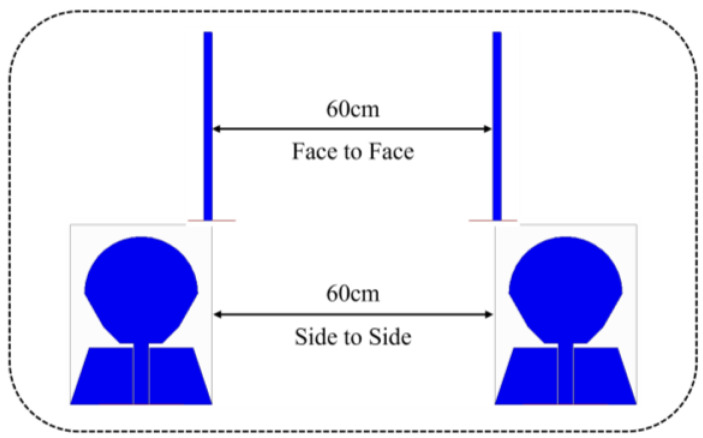
Measurement of antenna time-domain characteristics in HFSS.

As the proposed antenna is wideband, so its time-domain characterization becomes important [32]. In order to illustrate the time domain performance of the antenna, the time domain characteristics including group delay and transmission coefficient are investigated. Figure 29 shows the experimental set-up that uses twin antennas, where one is acting as a transmitter and another as receiver. These antennas are placed face-to-face and side-to-side at a distance of 60 cm, which is five times the wavelength of the lower operating frequency (around 2.5 GHz) in order to create far field atmosphere [33].

**Figure 30:**
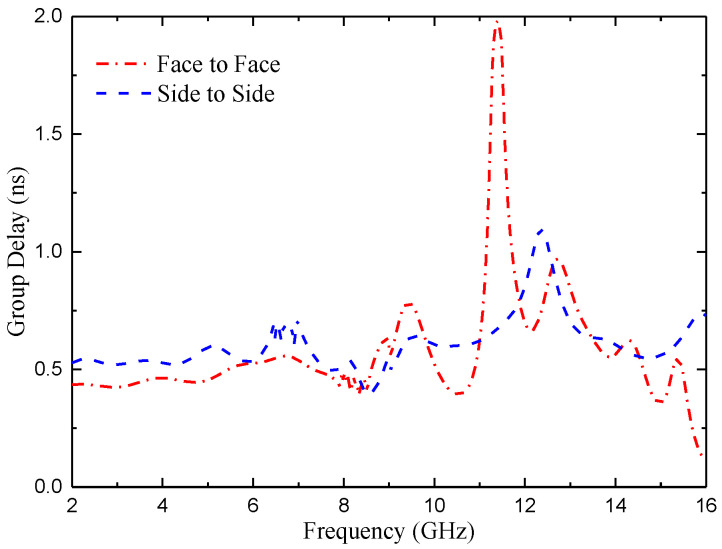
Group delay of the proposed antenna element.

The group delay of the antenna in the above two directions is shown in Figure 30. For a good wideband antenna configuration, the group delay should be minimal and constant throughout the operating bandwidth. It can be observed from the figure that in the operating band of the proposed antenna, the group delay remains stable at about 0.5 ns.

**Figure 31:**
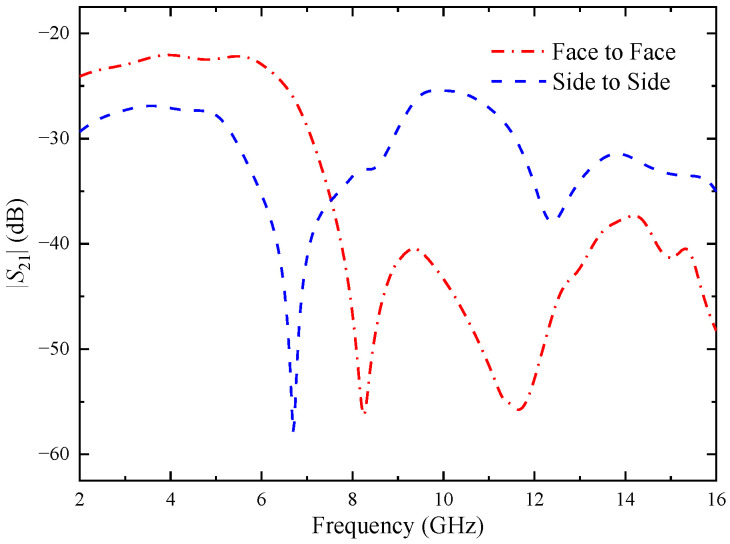
Transmission coefficients of the proposed antenna element.

The transmission coefficients of the antenna in both directions were simulated, as shown in Figure 31. It is verified that the transmission coefficient of the antenna in the whole working band is less than −22 dB.

In order to further verify the effectiveness of this design, the experimental results of the designed antenna are compared with those of the references. The comparative experimental results include antenna size, antenna impedance bandwidth, relative impedance bandwidth, number of MIMO antenna units, notch band, isolation, peak gain, and ECC. The details for each comparison item are shown in Table 2. In the references listed in Table 2, most antennas are not designed for notch and have low peak gain. The radiation efficiency of the antennas in [16,20] is also relatively low and the antenna in [14] has low isolation and a narrow impedance bandwidth. The sizes of the antennas in [20,34] are larger. By comparing the data in Table 2, it can be found that the designed antenna has a wide impedance bandwidth, with relative impedance bandwidth higher than 140%, low ECC, and good radiation efficiency. In a word, the antenna performance is better.

## 6. Conclusions

Two compact UWB–MIMO antennas with high isolation have been checked. One is a two-element MIMO antenna located parallel with an impedance bandwidth from 2.45–14.88 GHz, which is loaded with two RBs to achieve an isolation higher than 17 dB in the passband range. The other is a four-element MIMO antenna located at quadrature with an impedance bandwidth from 2.14–14.95 GHz. Through placing the antenna elements orthogonally and loading the cross branches, the antenna has an isolation degree of more than 20 dB in the passband range. Both can suppress the interference of WiMAX and ITU bands to UWB communication and are simple in structure and easy to process. At the same time, they have stable gain, much lower ECC (<0.02), DG close to 10 dB, TRAC below 10 dB, *MEG_ij_* below 3 dB, and CCL below 0.4 bit/s/Hz, which can be widely used in UWB communication systems.

## Figures and Tables

**Table 1:** Antenna geometry (unit: mm).

Parameter	*TL*	*FL*	*W*	*H*	*H*_1_	*W*_1_	*W*_2_	*W*_3_	*W*_4_
Size	38	68	68	1.6	7.6	9	16	4	11.4
Parameter	*W*_5_	*W*_6_	*W*_7_	*L*_1_	*L*_2_	*L*_3_	*L*_4_	*L*_5_	*L*_6_
Size	8	1.4	1.5	3.5	7	7.2	5.2	7	20
Parameter	*L*_7_	*S*_1_	*S*_2_	*S*_3_	*S*_4_	*g*	*G_h_*	*R*_1_	*H*_2_
Size	14	0.3	0.3	2	0.6	1	12	12	11

**Table 2:** Comparison of antennas in references and this paper.

Ref.	Size (mm^3^) (At Lowset Frequency)	Impedance Bandwidth (GHz)	Relative Bandwidth (%)	Number of Elements	Notch Band	Isolation (dB)	ECC	Gain (dBi)	Radiation Efficiency (%)
[12]	0.43*λ* × 0.26*λ* × 0.023*λ*	4.3–15.63	114	2	-	20	<0.0075	<5.35	>85
[14]	0.31*λ* × 0.31*λ* × 0.008*λ*	2.9–12	122	2	-	15	<0.02	<4.2	>60
[16]	0.27*λ* × 0.26*λ* × 0.016*λ*	2.9–12.2	123	2	-	17.8	-	<3.8	-
[17]	0.67*λ* × 0.67*λ* × 0.013*λ*	2.1–20	161	4	WiMAX	25	<0.02	<5.8	>80
[19]	0.43*λ* × 0.26*λ* × 0.016*λ*	3.2–12	115	4	-	22	<0.5	<4	>80
[20]	0.67*λ* × 0.67*λ* × 0.024*λ*	4.5–16.4	114	4	-	20	<0.002	<7.8	>61
[34]	0.73*λ* × 0.88*λ* × 0.003*λ*	3.89–17.09	126	4	-	15	<0.02	<6.8	>89
This work	0.57*λ* × 0.32*λ* × 0.013*λ*	2.45–14.88	143	2	WiMAX /ITU	>17	<0.02	<5.7	>82
	0.57*λ* × 0.57*λ* × 0.013*λ*	2.14–14.95	150	4	WiMAX /ITU	>20	<0.02	<5.9	>80

## Data Availability

Not applicable.

## References

[1] Mescia L., Mevoli G., Lamacchia C.M., Gallo M., Bia P., Gaetano D., Manna A. (2022). sinuous antenna for UWB radar applications. Sensors.

[2] Wang M., Tian X.W., Song L.Z. (2021). A ultra wideband dual-polarized antenna with high isolation degree for passive radar application. Electromagnetics.

[3] Mohammad K., Sajad M.A. (2021). Radar cross-section reduction of an UWB MIMO antenna using image theory and its equivalent circuit model. Int. J. RF Microw. Comput. Aided Eng..

[4] Emadian S.R., Ahmadi-Shokouh J., Ghobadi C., Nourinia J. (2018). Study on frequency and impulse response of novel triple band notched UWB antenna in indoor environments. AEU Int. J. Electron. Commun..

[5] Martínez-Lozano A., Blanco-Angulo C., García-Martínez H., Gutiérrez-Mazón R., Torregrosa-Penalva G., Ávila-Navarro E., Sabater-Navarro J.M. (2021). UWB-Printed rectangular-based monopole antenna for biological tissue analysis. Electronics.

[6] Kissi C., Särestöniemi M. (2020). Receiving UWB antenna for wireless capsule endoscopy communications. Prog. Electromagn. Res. C.

[7] Zhong Z.P., Liang J.J., Fan M.L., Huang G.L., He W., Chen X.C., Yuan T. (2019). A compact CPW-fed UWB antenna with quadruple rejected bands. Microw. Opt. Technol. Lett..

[8] Iqbal A., Smida A., Mallat N.K., Islam M.T., Kim S. (2019). A Compact UWB Antenna with Independently Controllable Notch Bands. Sensors.

[9] Liu J.B., Ding W.H., Chen J.H., Zhang A. (2019). New ultra-wideband filter with sharp notched band using defected ground structure. Prog. Electromagn. Res. Lett..

[10] Siddiqui J.Y., Saha C., Sarkar C., Shaik L.A., Antar Y. (2018). Ultra-wideband antipodal tapered slot antenna with integrated frequency-notch characteristics. IEEE Trans. Antennas Propag..

[11] Luo S., Chen Y., Wang D., Liao Y., Li Y. (2020). A monopole UWB antenna with sextuple band-notched based on SRRs and U-shaped parasitic strips. AEU Int. J. Electron. Commun..

[12] Mu W., Lin H., Wang Z., Li C., Yang M., Nie W., Wu J. (2022). A flower-shaped miniaturized UWB-MIMO antenna with high isolation. Electronics.

[13] Wang L.L., Du Z.H., Yang H.L., Ma R.Y., Zhao Y.C., Cui X.Q., Xi X.L. (2019). Compact UWB MIMO antenna with high isolation using fence-type decoupling structure. IEEE Antennas Wirel. Propag. Lett..

[14] Ren J., Hu W., Yin Y. (2014). Compact printed MIMO antenna for UWB applications. IEEE Antennas Wirel. Propag. Lett..

[15] Zhang S., Pedersen G.F. (2016). Mutual coupling reduction for UWB MIMO antennas with a wideband neutralization line. IEEE Antennas Wirel. Propag. Lett..

[16] Boumaaza K., Hebib S., Mouffok L. Compact two-port tapered microstrip feed MIMO antenna for UWB applications. Proceedings of the 2022 2nd International Conference on Advanced Electrical Engineering (ICAEE).

[17] Rekha V.S.D., Pardhasaradhi P., Madhav B.T.P., Devi Y.U. (2020). Dual band notched orthogonal 4-element MIMO antenna with isolation for UWB applications. IEEE Access.

[18] Agarwal S., Rafique U., Ullah R., Ullah S., Khan S., Donelli M. (2021). Double overt-leaf shaped CPW-Fed four port UWB MIMO antenna. Electronics.

[19] Srivastava G., Mohan A. (2016). Compact MIMO slot antenna for UWB applications. IEEE Antennas Wirel. Propag. Lett..

[20] Kumar P., Pathan S., Vincent S., Kumar O.P., Yashwanth N., Kumar P., Shetty P.R., Ali T. (2023). A compact quad-port UWB MIMO antenna with improved isolation using a novel mesh-like decoupling structure and unique DGS. IEEE T. Circuits II..

[21] Arumugam S., Manoharan S., Palaniswamy S.K., Kumar S. (2021). Design and Performance Analysis of a Compact Quad-Element UWB MIMO Antenna for Automotive Communications. Electronics.

[22] Kolangiammal S., Balaji L., Mahdal M. (2022). Design of compact planar monopole UWB MIMO antenna with four orthogonal elements and tapered fed configuration for wireless diversity applications. Electronics.

[23] He Z., Jin J. (2022). Compact quad-port MIMO antenna with ultra-wideband and high isolation. Electronics.

[24] Zhao X., Riaz S., Geng S. (2019). A reconfigurable MIMO/UWB MIMO antenna for cognitive radio applications. IEEE Access.

[25] Desai A., Kulkarni J., Kamruzzaman M.M., Hubalovsky S., Hsu H.T., Ibrahim A.A. (2022). Interconnected CPW Fed Flexible 4-Port MIMO Antenna for UWB, X, and Ku Band Applications. IEEE Access.

[26] Patel U., Upadhyaya T. (2022). Four-Port Dual-Band Multiple-Input Multiple-Output Dielectric Resonator Antenna for Sub-6 GHz 5G Communication Applications. Micromachines.

[27] Khan A.A., Jamaluddin M.H., Nasir J., Khan R., Aqeel S., Saleem J. (2016). Owais. Design of a dual-band MIMO dielectric resonator antenna with pattern diversity for Wimax and Wlan applications. Prog. Electromagn. Res. M.

[28] Rafique U., Agarwal S., Nauman N., Khalil H., Ullah K. (2021). Inset-fed Planar Antenna Array for Dual-band 5G MIMO Applications. Prog. Electromagn. Res. C.

[29] Kulkarni J., Sim C.Y.D., Desai A., Holdengreber E., Talware R., Deshpande V., Nguyen T.K. (2022). A Compact Four Port Ground-Coupled CPWG-Fed MIMO Antenna for Wireless Applications. Arab. J. Sci. Eng..

[30] Tang Z., Wu X., Zhan J., Hu S., Xi Z., Liu Y. (2019). Compact UWB-MIMO Antenna With High Isolation and Triple Band-Notched Characteristics. IEEE Access.

[31] Khangarot S., Sravan B.V., Aluru N., Mohammadsaadh A.W., Poonkuzhali R., Kumar O.P., Ali T., Manoharapai M.M. (2020). A compact wideband antenna with detailed time domain analysis for wireless applications. Ain Shams Eng. J..

[32] Addepalli T., Desai A., Elfergani I., Anveshkumar N., Kulkarni J., Zebiri C., Rodriguez J., Abd-Alhameed R. (2021). 8-Port Semi-Circular Arc MIMO Antenna with an Inverted L-Strip Loaded Connected Ground for UWB Applications. Electronics.

[33] Cruz J.D.N., Serres A.J.R., de Oliveira A.C., Xavier G.V.R., de Albuquerque C.C.R., da Costa E.G., Freire R.C.S. (2019). Bio-inspired Printed Monopole Antenna Applied to Partial Discharge Detection. Sensors.

[34] Lin G.S., Sung C.H., Chen J.L., Chen L.S., Houng M.P. (2016). Isolation improvement in UWB MIMO antenna system using carbon black film. IEEE Antennas Wirel. Propag. Lett..

